# Molecular Diagnosis and Phylogenetic Analysis of Hemotropic Mycoplasmas and *Anaplasma* spp. in Peripheral Blood of Goats in Northern Iran and Their Hematobiochemical Alterations

**DOI:** 10.1155/vmi/5985068

**Published:** 2026-06-30

**Authors:** Masoud Nematinezhad, Hassan Sharifiyazdi, Saeed Nazifi, Tina Yaghoobpour, Mojtaba Rahsepar, Seyed Mohammad Bagher Hosseini

**Affiliations:** ^1^ Department of Clinical Sciences, School of Veterinary Medicine, Shiraz University, Shiraz, Iran, shirazu.ac.ir

**Keywords:** *Anaplasma*, goat, Mazandaran, *Mycoplasma*, phylogenetic analysis

## Abstract

*Anaplasma* and hemoplasma are hemotropic bacteria that infect erythrocytes of various animal hosts and are responsible for significant veterinary health problems. This study aimed to determine the prevalence of *Anaplasma* spp. and hemotropic *Mycoplasma* spp. in goats in Mazandaran Province, Northern Iran. A total of 149 blood samples were collected from goats of different ages and sexes during the spring season. The samples were analyzed using polymerase chain reaction (PCR) to identify *Anaplasma* spp. and *Mycoplasma* spp. To identify bacterial species, representative amplicons of *Mycoplasma* (*n* = 4) and *Anaplasma* (*n* = 6) were sequenced. PCR testing revealed a prevalence of 19.4% for *Anaplasma* spp. and 28.1% for hemotropic *Mycoplasma*. Statistical analysis of blood parameters indicated significant differences between the *Anaplasma*‐positive and negative groups, with a notable decrease in red blood cell (RBC) count and hematocrit (HCT) levels, while alkaline phosphatase (ALP) and triglyceride levels showed significant increases. In contrast, no significant differences were observed in hematological and biochemical parameters for the *Mycoplasma*‐positive group. Molecular and phylogenetic analyses confirmed the presence of *M. ovis* (PQ793282), *Anaplasma capra* (PV176424), *Anaplasma ovis* (PV185359), and *Anaplasma phagocytophilum* (PV175399) in Iranian goats. This research represents the first report of *M. ovis* and *A. capra* in Iran and highlights the need for enhanced molecular surveillance of *Anaplasma* and *Mycoplasma* infections in livestock. Consequently, these pathogens—especially those exhibiting close genetic relationships with human pathogens—represent significant threats to both livestock and human health. This underscores the need for enhanced surveillance, recognition of potential zoonotic risks, vector control strategies, and vaccination measures.

## 1. Introduction

Food safety is a major focus of governmental programs. In Iran, a substantial number of livestock products are lost each year for various reasons, with infectious diseases—particularly those caused by hemotropic bacteria—being among the most significant factors [1, 2]. According to reports from the Food and Agriculture Organization (FAO), infectious diseases represent a major constraint to global livestock production, exerting considerable economic impact on animal health and productivity. These losses are generally more pronounced in developing and low‐income countries compared with developed regions [3]. *Anaplasma* is a genus in the Anaplasmataceae family, consisting of Gram‐negative, intracellular bacteria that infect the blood cells of mammals [4, 5]. Species of *Anaplasma* include *A. marginale*, *A. bovis*, *A. ovis*, *A. centrale*, *A. platys*, and *A. phagocytophilum*, which infect a variety of mammalian hosts [5]. These pathogens are transmitted through tick vectors, with significant genera including *Rhipicephalus, Ixodes, Amblyomma,* and *Dermacentor* [4–6]. Transmission occurs mechanically through biting flies or blood‐contaminated instruments, as well as biologically by ticks [7]. Anaplasmosis, characterized as hemolytic anemia, is globally distributed and endemic in many tropical and subtropical regions [8, 9]. *Eperythrozoon ovis*, currently named *Mycoplasma ovis*, is found in small ruminants globally, albeit less frequently in goats [10]. Initially classified as a *Rickettsia* in the Anaplasmataceae family, it was first reported by Neitz in 1937 [11] as round to ovoid, comma, and dumbbell‐shaped bodies measuring 0.5–1 μm, adhering to the surface of erythrocytes. Two hemoplasma species can infect small ruminants (sheep and goats), namely, *Candidatus Mycoplasma haemovis* and *Mycoplasma ovis* [10]. Hemoplasmas are small, pleomorphic bacteria that attach to red blood cell (RBC) surfaces, causing hemolytic anemia, characterized by a reduction in circulating RBCs and decreases in hemoglobin and hematocrit (HCT) levels, alongside hemosiderin deposition in tissues [12, 13]. While sheep exhibit pronounced hemolytic anemia due to *Mycoplasma ovis*, goats may act as latent carriers [14, 15]. This pathogen is zoonotic, posing potential risks to humans, deer, and reindeer [10], and can lead to decreased milk production, increased lamb mortality, and poor weight gain [16]. *Mycoplasma ovis* infections have been recorded across all continents, particularly in Malaysia, Japan, China, and the Philippines [13]. Transmission vectors include various blood‐sucking arthropods, such as *Rhipicephalus bursa*, *Haemaphysalis plumbeum*, *Tabanus bovinus*, *Stomoxys calcitrans*, *Haematobia irritans*, and *Aedes camptorhynchus* [14, 17].

The polymerase chain reaction (PCR) method is preferred for diagnosing anaplasmosis and mycoplasmosis, offering greater specificity and sensitivity compared to traditional diagnostic techniques [18].

Given the economic importance of goat production in Iran, a comprehensive understanding of the accurate prevalence and genetic diversity of *Anaplasma* and hemotropic *Mycoplasma* species is crucial in endemic areas. Currently, there is a significant lack of data regarding the specific causative agents of caprine hemoplasma infections and anaplasmosis implicated in infections within Iran. The zoonotic significance of *Anaplasma phagocytophilum* has recently been revealed, leading to increased interest in these organisms [19, 20]. Furthermore, a likely novel tick‐transmitted *Anaplasma* species was recently detected in asymptomatic goats in China and has been acknowledged as a source of human illness; the provisional name “*Anaplasma capra*” was offered [21]. This suggests that the agent may represent a significant public health threat and could be implicated in cases of anaplasmosis of unknown etiology [22, 23]. A meta‐analysis conducted in 2023 revealed that the average prevalence of *A. capra* is 5.9% in humans, 11.3% in animals, and 7.8% in ticks [24].

The primary objective of this study was to find out the prevalence of *Anaplasma* spp. and *Mycoplasma* spp. infections in goat populations in Mazandaran Province, Northern Iran, using PCR techniques followed by phylogenetic analyses to explore the genetic relationships among the strains identified. Additionally, the study aims to assess the hematological and biochemical alterations in infected goats to realize the significant health impacts associated with these infections.

## 2. Materials and Methods

### 2.1. Sample Collection and DNA Extraction

**FIGURE 1:**
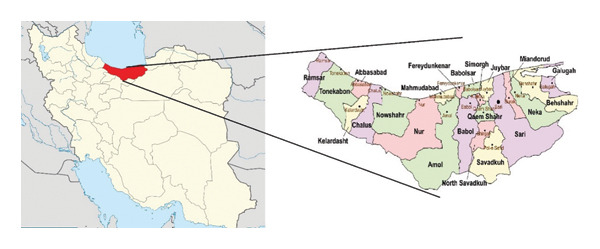
Map of the study area. Mazandaran Province of Northern Iran, shown in color on the map, is where the samples were taken.

Blood samples were randomly taken from 149 clinically healthy goats across multiple localities in Northern Iran (Figure 1). This cross‐sectional survey was executed in April 2024 in Mazandaran Province, Iran. The area (36° 33′N 52° 40′E) has a hot‐summer Mediterranean climate, with an average annual temperature of 17°C and over 1000 mm of yearly precipitation [25]. Samples were collected from the jugular vein and stored in both EDTA and non‐EDTA tubes. A comprehensive blood count was performed with a Veterinary Hematology Analyzer (Nihon Kohden, MEK‐6450 Celltac Alpha, Tokyo, Japan). Standard blood smears were made, and the residual samples were allocated for DNA extraction. Genomic DNA was extracted from 200 μL of whole blood utilizing a commercial Blood Genomic DNA Purification Kit (Parstous, Iran). The purity and concentration of DNA were evaluated using a NanoDrop spectrophotometer (Thermo Fisher Scientific, NanoDrop, USA), and the extracted DNA was preserved at −20°C until subsequent analysis.

### 2.2. Measurement of Biochemical Parameters

Serum biochemical parameters, such as blood urea nitrogen (BUN), creatinine, glucose, cholesterol, triglycerides (TGs), total protein, albumin, calcium, phosphorus, magnesium, iron, AST, ALT, alkaline phosphatase (ALP), GGT, CPK, and LDH were quantified utilizing commercial kits (Pars Azmoon Co., Tehran, Iran) and a biochemical autoanalyzer (Alpha Classic AT++, Sanjesh, Iran). Globulin values were determined by subtracting albumin values from total protein. Serum sodium and potassium levels were measured utilizing a flame photometer (Fater Electron Company, Tehran, Iran).

#### 2.2.1. Molecular Diagnosis Method

Following the protocols described by Hoelzle et al. [26] and Seja et al. [27] for *Mycoplasma* detection and established procedures for *Anaplasma* detection, a PCR assay was performed to identify hemoplasmas and anaplasmas. For hemoplasmas, an approximately 1000‐bp fragment of the 16S rDNA gene was amplified using the universal primer pair 16S_HAEMOforw (5′‐GGCCCATATTCCTRCGGGAAG‐3′) and 16S_HAEMOrev (5′‐ACRGGATTACTAGTGATTCCA‐3′). For *Anaplasma* spp., an ∼806‐bp region of the 16S rDNA gene was amplified using the primer pair 8F (5′‐AGAAGAAGTCCCGGCAAAC‐3′) and 8R (5′‐GAGACGACTTTTACGGATTAGCTC‐3′). In addition, for the detection of Piroplasma, an approximately 420‐bp fragment of the small subunit ribosomal RNA (SSU rRNA) gene was amplified using the primers FPIR (5′‐CTAAGAATTTCACCTCTGACAGT‐3′) and RPIR (5′‐GACACAGGGAGGTAGTGACAAG‐3′) [28].

PCR amplification was performed in a final reaction volume of 25 μL using a 2× Red PCR Master Mix (Ampliqon, Denmark). Each reaction contained 12.5 μL of 2× master mix, 1 μL of each primer (10 pmol/μL), 2 μL of template DNA (∼50 ng), and nuclease‐free water to reach the final volume. The final concentrations in the reaction were 1.5 mM MgCl_2_ and 0.2 mM of each dNTP. To ensure the reliability of the results, a positive control and a negative control were included in every PCR run to monitor for potential contamination and assay performance.

DNA amplification for all *Anaplasma* and hemoplasma assays was performed using a uniform thermocycling protocol consisting of an initial denaturation at 94°C for 3 min, followed by 35 cycles of denaturation at 94°C for 50 s, annealing for 45 s (56°C for *Anaplasma* spp. and 58°C for *Mycoplasma* and *Piroplasma*), and extension at 72°C for 45 s, with a final extension at 72°C for 7 min.

The PCR products were analyzed by electrophoresis on a 1.5% agarose gel at 100 V for 60 min. The gel was stained with 5% Red Safe in‐gel stain, and the bands were visualized under ultraviolet light using a gel documentation system (UV Tech Transilluminator, USA).

### 2.3. DNA Sequencing and Phylogenetic Analyses

To ensure accurate species identification, representative PCR products positive for *Mycoplasma* and *Anaplasma* were sent to Pishgam Biotech (Tehran, Iran) for sequencing. The resulting sequences were compared with sequences available in the NCBI database using the BLAST search tool. The Clustal W program, implemented in MEGA software (Version 11), was used to align the obtained sequences with homologous sequences from the GenBank database. The Kimura 2‐parameter distance estimate was used for phylogenetic analyses, and phylogenetic trees were constructed using the maximum likelihood method [29].

### 2.4. Statistical Analysis

All data were analyzed with SPSS Version 22. A *p* value below 0.05 was considered statistically significant. The independent sample *t*‐test and Mann–Whitney test were employed for parametric and nonparametric datasets, respectively. Animals exhibiting no clinical indications and possessing normal blood values were classified as the control group.

## 3. Results

**FIGURE 2:**
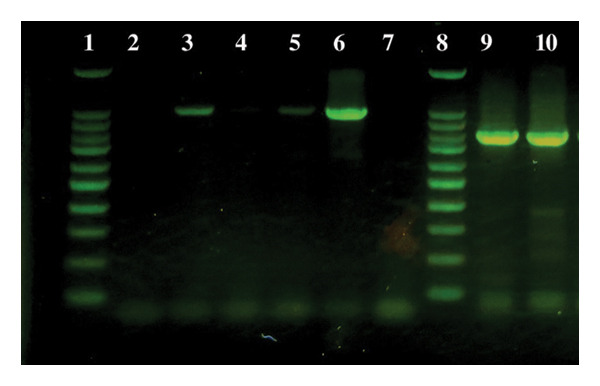
Agarose gel electrophoresis of PCR products targeting the 16S rDNA region of *Anaplasma* spp. and *Mycoplasma ovis*. Lanes 1 and 8: 100‐bp DNA ladder; lanes 3 and 5: field samples positive for *M. ovis*; lane 6: positive control for *M. ovis*; lane 4: negative control for *M. ovis* (nuclease‐free water); lane 9: field sample positive for *Anaplasma* spp.; lane 10: positive control for *Anaplasma* spp.; lane 7: negative control for *Anaplasma* spp. (nuclease‐free water).

Agarose gel electrophoresis in Figure 2 shows DNA bands from PCR amplification of the 16S rDNA gene of *Anaplasma* spp. and *Mycoplasma ovis*. PCR analysis of 149 goat blood samples revealed that 29 samples (5 males, 24 females) were positive for *Anaplasma* spp., 42 samples (5 males, 37 females) were positive for hemotropic *Mycoplasma*, and 13 samples (2 males, 11 females) showed coinfection with both *Anaplasma* and *Mycoplasma*. These results indicate a prevalence of 19.4% for *Anaplasma* and 28.1% for *Mycoplasma ovis* in goats from Mazandaran Province, Northern Iran. These positive samples originated from various farms in different regions of Mazandaran Province, Northern Iran (Figure 1).

**FIGURE 3:**
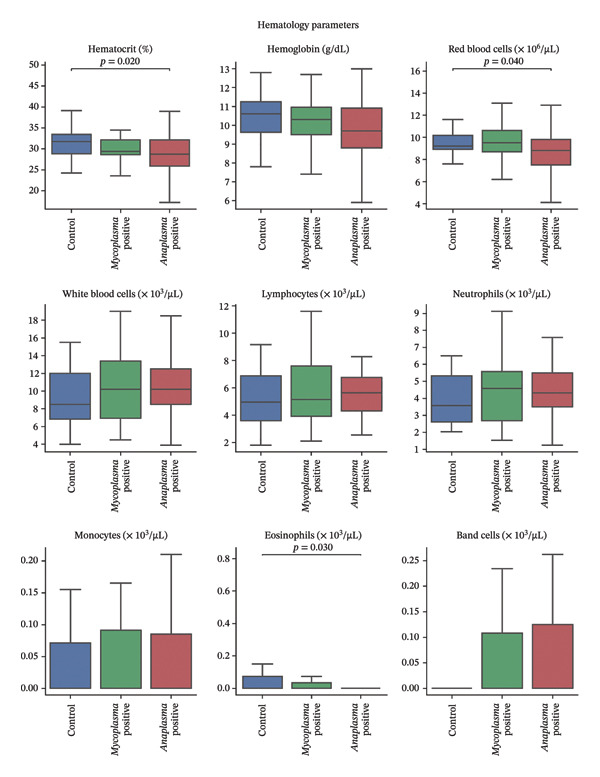
The comparison of hematological values of PCR‐positive and PCR‐negative goats (for *Anaplasma* spp. and *Mycoplasma ovis*).

**FIGURE 4:**
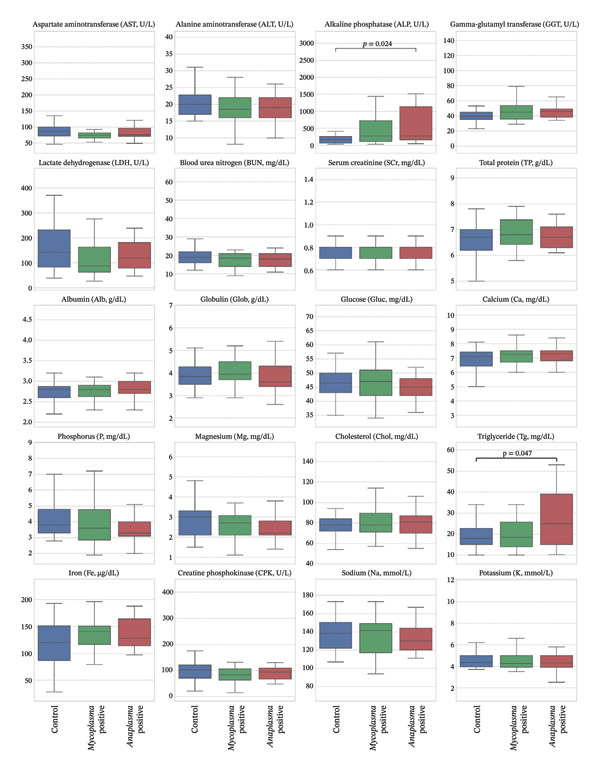
The comparison of biochemical values of PCR‐positive and PCR‐negative goats (for *Anaplasma* and *Mycoplasma* spp.).

Statistical analysis revealed a significant difference between the *Anaplasma*‐positive group and the negative group in RBC counts (× 106 cells/μL) (*p* = 0.042) and HCT (%) (*p* = 0.026) for hematological parameters, as well as in TG (mg/dL) (*p* = 0.047) and ALP levels (*p* = 0.01) for biochemical parameters. No significant differences were observed between the *Mycoplasma* group and the negative group in either hematological or biochemical parameters (*p* > 0.05) (Figures 3 and 4). Furthermore, there was no significant relationship between *Anaplasma* infection and the age or sex of the goats (*p* > 0.05), nor was there a significant relationship between *Mycoplasma* infection and age or sex (*p* > 0.05).

To ensure accurate identification of the hemotropic bacterial species, representative amplicons of *Mycoplasma* (*n* = 4) and *Anaplasma* (*n* = 6) originating from positive farms were randomly sequenced and compared to available data in the GenBank database. All four samples submitted for *Mycoplasma* spp. sequencing were identified as *Mycoplasma ovis*. Sequence analysis revealed 100% similarity across all four isolates. Additionally, of the six samples sent for *Anaplasma* spp. sequencing, four were identified as *Anaplasma ovis* (exhibiting 100% similarity to each other), one as *Anaplasma phagocytophilum*, and one as *Anaplasma capra.* GenBank accession numbers PQ793282, PV176424, PV185359, and PV175399 were provided in the NCBI database for Iranian *M*. *ovis*, *A*. *capra*, *A*. *ovis*, and *A*. *phagocytophilum* strains, respectively.

**FIGURE 5:**
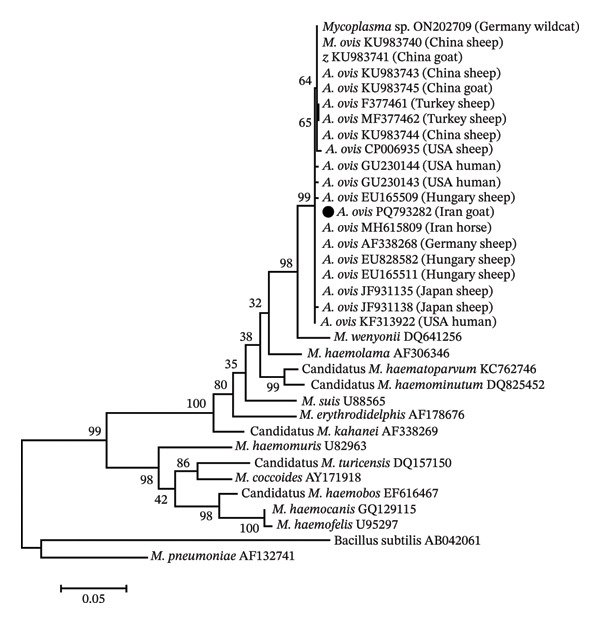
Molecular phylogenetic analysis of *Mycoplasma* sp. based on partial 16S rRNA gene sequences. The tree was constructed using the maximum likelihood (ML) method in MEGA X, based on a total alignment length of approximately 1000 bp. The Iranian strains obtained in this study are highlighted with black circles (·). Numbers at the nodes represent bootstrap confidence values (percentage of 1000 replicates); only values of taxonomic relevance are shown. The scale bar indicates the number of nucleotide substitutions per site.

The phylogenetic study of *M*. *ovis* isolates from goats in Iran indicated a close genetic affinity with previously documented *M. ovis* strains in Iranian horses. Also, the phylogenetic tree (Figure 5) illustrates that the Iranian *M. ovis* isolates are closely related to strains from sheep, goats, and humans in China, Turkey, Hungary, and the USA.

The Iranian isolates formed a separate branch alongside *M. ovis* sequences from Iranian sheep (MH615089) and goats (GU230143), suggesting a common ancestry and possible interspecies transmission.

**FIGURE 6:**
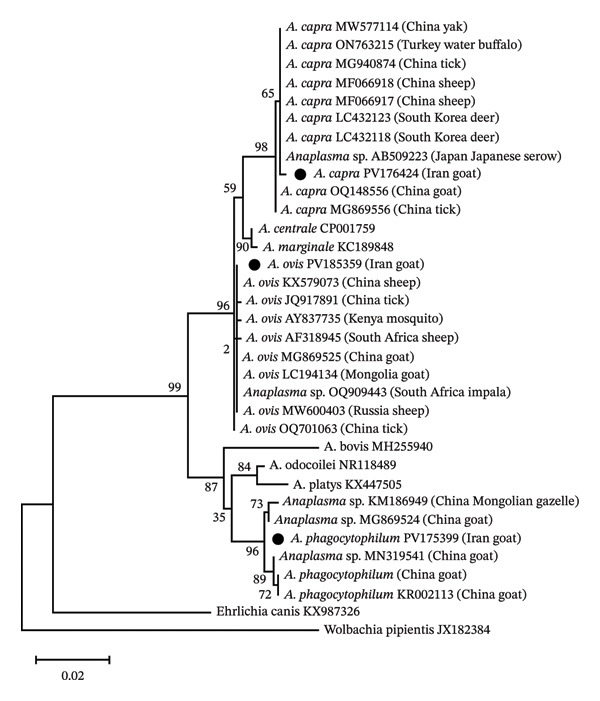
Molecular phylogenetic analysis of *Anaplasma* spp. based on partial 16S rRNA gene sequences. The tree was constructed using the maximum likelihood (ML) method in MEGA X, based on a total alignment length of approximately 806 bp. The Iranian strains obtained in this study are highlighted with black circles (·). Numbers at the nodes represent bootstrap confidence values (percentage of 1000 replicates); only values of taxonomic relevance are shown. The scale bar indicates the number of nucleotide substitutions per site.

The phylogenetic analysis of *A*. *capra* isolates from Iranian goats indicated their genetic similarity with other *Anaplasma* strains from various hosts and geographical areas. The phylogenetic tree (Figure 6) illustrates that the Iranian *A. capra* isolate (PV176424) is closely related to other *A*. *capra*. strains, including those from Japan (Japanese serow), Turkey (water buffalo), and China (yak, sheep, and tick). The robust bootstrap support values (between 65 and 99) reflect the reliability of these clades.

The Iranian *A. capra* isolate exhibited the greatest similarity to the Japanese and Chinese strains, indicating a potential transboundary transmission route or a shared evolutionary ancestor. The phylogenetic analysis underscores the host range of *A*. *capra*, suggesting its potential for interspecies transmission in domestic and wild ruminants.

The phylogenetic tree indicates that *A. capra* forms a separate branch from *A. ovis*, a similar pathogen commonly found in sheep and goats, in multiple locations, including Iran, Russia, Mongolia, and China. This differentiation confirms the genetic divergence between the two species, despite their occasional presence in the same host species.

The Iranian *A. ovis* isolates are closely aligned with strains from Russia and South Africa, suggesting a wider geographical distribution and potential migration of infected animals or vectors.

## 4. Discussion

*Mycoplasma* spp. and *Anaplasma* spp. are hemotropic bacteria that cause significant economic losses in livestock [9, 13]. Numerous investigations have been conducted on these bacteria to mitigate and control their impact; however, identifying and diagnosing them in goats remain challenging [4, 12–14]. The chronic nature of *Mycoplasma* infections in goats often leads to asymptomatic carriers, allowing for transmission to other animals and possibly humans, complicating hygienic management practices [4, 9, 13]. Consequently, the efforts focused on detecting, diagnosing, and controlling these infections are critical.

This study employed PCR and paraclinical findings, revealing positive infections for both *Anaplasma* and *Mycoplasma* in apparently healthy goats. Our findings demonstrated that 19.4% of all samples tested positive for *Anaplasma* (95% CI: 13.44%–26.74%) and 28.1% (95% CI: 20.96%–35.41%) for *Mycoplasma*. Importantly, *Mycoplasma ovis*, which infects both sheep and goats in regions with varying temperatures—including tropical and subtropical areas—poses a significant risk [13]. Notably, the climate of Northern Iran, particularly Mazandaran, provides an ideal environment for a diverse range of tick species capable of transmitting hemotropic bacteria in goats [5, 30].

According to our study, this is the first report of *M. ovis* in goats in Iran, with a prevalence of 6.77%. In previous work, Kalantari et al. [31] provided the first molecular evidence of *M. ovis* in horses in Iran, associated with mild anemia due to decreased HCT and hemoglobin concentration, as well as RBC count. Our sequencing results align with those of Kalantari and colleagues, suggesting interspecies transmission. The significant genetic similarity across *M. ovis* isolates from goats, sheep, horses, wildcats, and humans supports the possibility of cross‐species transmission. This finding highlights the necessity for ongoing molecular surveillance to track the dissemination of *M. ovis* and its zoonotic potential.

Furthermore, our study found that *Anaplasma* spp. and *Mycoplasma ovis* were prevalent in goat blood samples in Northern Iran, with hematological and biochemical changes particularly attributed to *Anaplasma* infection. The statistical analysis highlighted marked alterations in hematological parameters, specifically a decrease in RBC count and HCT among the *Anaplasma*‐infected group. This was accompanied by significant increases in biochemical markers like TGs and ALP. In contrast, no significant alterations were detected in the *Mycoplasma*‐infected group. Analysis of the 18S ribosomal region to detect Piroplasma (*Theileria luwenshuni, Babesia odocoilei, Babesia capreoli,* and *Babesia aktasi*) in goats yielded negative results, suggesting that the observed hematological and biochemical alterations were not attributable to infection with these hemotropic agents in this study.

Moreover, a statistically significant correlation was observed between *Anaplasma*‐positive cases and the biochemical variables ALP and TGs in dairy cows. Elevated liver enzyme levels in the bloodstream indicate liver function. Alekish et al. found increased levels of ALT, GGT, ALP, LDH, and AST; however, only LDH and AST levels exhibited statistically significant increases. This is consistent with findings in spontaneously infected dairy cows with nonzoonotic hemotropic infections such as *Anaplasma marginale* [32]. The increased AST and LDH levels may result from hepatic injury in chronically affected cows, also stemming from cardiac or erythrocytic injury. Ganguly et al. similarly reported elevated serum AST and ALT values [33].

Interestingly, *Anaplasma* infections have varying effects on blood cholesterol and TG levels. While Ahmadi‐Hamedani et al. reported no significant change in TG and cholesterol levels between healthy and diseased goats [34], Khaki et al. discovered a substantial decrease in cholesterol levels in infected goats infected with *A. ovis* [35]. Additionally, a study by Messick [36] noted a significant increase in TG levels and a marked decrease in cholesterol levels in infected goats, linking increased TG levels to adipose tissue breakdown that triggers hepatic TG production. Furthermore, liver damage may disrupt normal cholesterol synthesis, contributing to lower total cholesterol levels.

Emerging zoonotic infections, such as *Mycoplasma* spp., pose significant and uncertain health risks to both humans and animals worldwide [36]. The absence of effective in vitro growth methods for reliably isolating these bacteria presents substantial challenges for research. In a study by Maggi et al. [37], the most commonly identified *Mycoplasma* spp. was found to be similar to *Mycoplasma ovis*, a bacterium typically associated with ruminants such as sheep, goats, deer, and Japanese serows [38, 39]. Notably, while *M. ovis* is frequently found in these species, it has been less studied in humans. The first documented human infection involving a *Mycoplasma ovis*–like bacterium was reported by Sykes et al. [40]. The infected individual, a veterinarian, was also diagnosed with an infection from *Bartonella henselae*. This case underscores the potential for zoonotic transmission of *Mycoplasma* spp., particularly among individuals in close contact with infected animals.

Sequencing of positive samples revealed a close genetic relationship between Iranian isolates and strains from other countries reported from humans, other livestock, and wildlife animals, emphasizing the need for continuous surveillance. These results underscore the genetic diversity of *Anaplasma* species in Iran and their potential zoonotic and transboundary implications. The phylogenetic tree encompasses *A. phagocytophilum* and additional *Anaplasma* species, establishing distinct clades that affirm their genetic divergence from *A. capra* and *A. ovis*. The close genetic affinity between the Iranian *A. capra* and strains from remote areas highlights the necessity for ongoing surveillance and molecular characterization to track the dissemination and evolution of these diseases.

The current study estimates the pooled prevalence rate of *Anaplasma* spp. infection in Mazandaran, Iran, at 19.4%. Across different regions in Iran, prevalence rates vary: Ahvaz (54%) [41, 42], Mashhad (46%) [43], Gonabad and Mashhad (45%) [34], Mazandaran (37%) [30], Isfahan (28%) [44], Kerman (3%), and Hamedan (1%) [45]. The highest prevalence is observed in Southwestern and Northeastern Iran.

In the Middle East, *Anaplasma* spp. infections have been reported in Jordan, Egypt, UAE, Iraq, Qatar, Cyprus, and Israel [20, 46–48]. In Russia, prevalence among herbivores ranged from 0.5% to 1% [49]. In Pakistan, 5.81% of blood samples tested positive for *Anaplasma* [50]. In Turkey, 9% of bovines tested positive via PCR [6], with comparable environmental conditions in Western Iran. In Iraq, *Anaplasma* spp. was found in cattle, sheep, goats, and ibex [46]. The prevalence in Pakistan is similar to that in Khorasan Razavi, Iran. Additionally, molecular analyses at the Iran–Afghanistan border (2013–2014) detected *Anaplasma* DNA in 26.4% of samples [47]. Variations in prevalence may be due to differences in sampling methods, geography, climate, and vector presence.

Previous studies indicated varying prevalence rates of *Mycoplasma ovis* in sheep, with reports of 79.5% in Southeastern Australia, 12.8% in northern regions, 49% in Tasmania, and 6.3% in Tunisia [51–53].

Based on PCR analysis, *Anaplasma ovis* prevalence in goats was reported as 76% in Africa, with a 53% prevalence [54–56]. Previous findings suggest that PCR is more sensitive than microscopic evaluation for detecting blood pathogens [41]. Misinterpretation may occur due to similarities with Howell–Jolly bodies, artifacts, or other intra‐RBC [4, 57]. Furthermore, instances of ELISA‐negative yet PCR‐positive samples may denote recently infected goats that have not yet seroconverted or exhibit a weak immune response [4, 58]. Conversely, cases with PCR‐negative but ELISA‐positive results may indicate chronic phases of anaplasmosis. Therefore, PCR testing offers a more reliable detection method [59].

Challenges in determining the number of infected goats can arise from husbandry practices. Owners may not perceive any goats as unhealthy, complicating assessments of production loss. Variations in mean packed cell volume (PCV) among studies may be attributed to water accessibility [4]. Some owners provide water once a day, while others offer unlimited access, affecting health outcomes; for instance, the reported mean PCV levels were 24.5% and 25.1% in the investigations by Adogla‐Bessa and Aganga [60], while Soosaraei et al. [4] reported 33% in healthy goats in Botswana. Overall, factors such as flock size, climate, *Anaplasma* strain, livestock husbandry practices, grazing systems, and tick abundance influence infection prevalence [19, 61]. The study conducted by Barbosa et al. [62] found that 51% of goats tested were anemic, which poses a significant challenge to animal production. For an extended duration, gastrointestinal (GI) parasites, particularly *Haemonchus contortus*, have been associated with anemic crises in small ruminants. Various studies have indicated a link between tick‐borne diseases and anemia [63–66].

This study has certain limitations, such as the lack of detailed information on all *Anaplasma* species detected. Future research should adopt the RFLP‐PCR method for species identification and investigate other genetic markers of this agent to gain a comprehensive understanding of its genetic diversity and epidemiology. In addition, sampling was conducted during a single season (spring). Because tick‐borne infections such as anaplasmosis show marked seasonal variation associated with vector activity, the prevalence reported here may not fully represent the annual epidemiological pattern in goats in Mazandaran Province. Longitudinal studies including multiple seasons are therefore recommended to provide a more comprehensive epidemiological assessment.

Anaplasmosis remains one of the most notable endemic infections in several regions of Iran, exhibiting seasonal variability. It is notably more prevalent during the summer and spring months in Mashhad, Kurdistan, West Azerbaijan, Kerman, Mazandaran, Ahvaz, Isfahan, Hamedan, and Gonabad [5, 30, 34, 67–69]. The prevalence of *Anaplasma* disease has been reported as 34% in domestic ruminants, with Khozestan exhibiting the highest contamination rate at 54%, while Hamedan reported the lowest at 1% [5]. Our study indicated that 19.4% of the goat population in Mazandaran tested positive for *Anaplasma* spp. during the spring season, aligning with seasonal trends and emphasizing the need for focused control measures during spring and summer, when tick activity peaks.

This study is the first to report on *M. ovis* in goats in Iran, providing valuable data on its prevalence and the relationship between positive samples and changes in hematological and biochemical factors. Further investigations are required to determine the presence of this infection in other regions of Iran. The phylogenetic proximity of the Iranian goat isolates to *M. ovis* strains from humans in the USA indicated a potential zoonotic risk, highlighting the public health implications related to this hemoplasma. The lack of hematological changes and absence of anemia could be attributed to the chronic phase of the disease at the time of blood collection. Future studies should examine the mechanisms underlying cell‐mediated immune responses and conduct genomic analyses to evaluate the zoonotic potential of this infection. Ultimately, enhanced surveillance and control measures for these two infections are warranted. Given the limited knowledge of the socioeconomic impacts of these infections in goats, improving diagnostic tools, implementing vaccination strategies, and utilizing effective pesticides to reduce insect populations are recommended strategies.

Pathogens can be found in wild animals, as they serve as reservoirs [70, 71]. A major concern for public health and the cattle industry is the emergence of infectious disease pathogens with a wildlife origin [72–74]. The recent isolation of *A*. *capra* from Chinese patients exhibiting vague symptoms, along with the potential for central nervous system (CNS) complications, raises serious concerns about the species’ impact on public health [21, 75].

At the Chungbuk Wildlife Center in Korea, Amer et al. [76] detected *A. capra* DNA in blood samples at a rate of 17.7%. Satow et al. reported similar findings in Japanese serow populations, as well as in yaks from China, ticks in China, and deer and sheep in South Korea [77]. However, there is a dearth of epidemiological data regarding *A*. *capra* in Iranian wildlife. It appears that *A. capra* has a wide host range, as indicated by low infection rates in cattle, sheep, and goats across Korea, China, and Sweden [22, 78–80].

Investigating wildlife as reservoirs of infections is essential for understanding zoonotic diseases and their potential effects on public health and the cattle industry. Wildlife frequently harbors pathogens such as *A*. *capra*, which can transfer to humans and domestic animals, posing significant health threats [22]. Research has demonstrated the presence of *A. capra* in various wildlife species across regions like Korea, China, and Japan [76]. Nevertheless, information regarding its prevalence in Iranian wildlife remains scarce. This knowledge gap is concerning, as wildlife can act as reservoirs for infections that may then infect livestock, leading to economic losses in agriculture. Monitoring wildlife populations is crucial for early detection and management, particularly in regions with limited data, to avert the transmission of zoonotic diseases. Consequently, focusing research efforts on wildlife as potential reservoirs is essential for mitigating health risks and protecting both human and animal populations.

Given the influence of climate change on tick vector behavior and ecosystems, the likelihood of human, livestock, and wildlife animals’ exposure to pathogens derived from this host species is increasing. On the other hand, investigating wildlife as reservoirs of zoonotic diseases is critical for public health and livestock management. Therefore, the development of health policies and integrated management strategies is essential to mitigate these risks and ensure food security. Additionally, multidisciplinary research that brings together wildlife biologists, veterinarians, epidemiologists, and public health experts is necessary to advance our understanding of these pathogens. Utilizing molecular techniques such as DNA sequencing, bioinformatics, and RFLP‐PCR can further enhance the accuracy of hemotropic agent identification and tracking within different populations.

## 5. Conclusion

This study marks the first reported incidence of *M. ovis* and *A*. *capra* in Iran. The presence of various hemotropic bacterial species in goats underscores the challenges associated with diagnosing and controlling these infections, as infected animals may act as asymptomatic carriers, facilitating disease transmission to other livestock and potentially to humans. The zoonotic potential of these pathogens is particularly concerning for individuals with close contact with animals. The findings highlight the urgent need for enhanced surveillance and control measures, including the development of improved diagnostic tools, effective zoonotic risk management, vector control strategies, and preventive programs.

## Author Contributions

The study conception and design were performed by Hassan Sharifiyazdi and Saeed Nazifi. Blood sampling and PCR analysis in this study were performed by Masoud Nematinezhad and Tina Yaghoobpour. Measurement of the biochemical and hematological parameters was performed by Saeed Nazifi, Mojtaba Rahsepar, and Seyed Mohammad Bagher Hosseini. Phylogenetic analysis was performed by Hassan Sharifiyazdi. The first draft of the manuscript was written by Tina Yaghoobpour and Masoud Nematinezhad, and all authors made comments on the manuscript.

## Funding

This work was financially supported by Shiraz University, grant number 2GRC1M143807.

## Ethics Statement

This study was approved by the Committee of Research and Law for Animal Experiments of School of Veterinary Medicine, Shiraz University (approval number 2GCB1M143807).

## Conflicts of Interest

The authors declare no conflicts of interest.

## Data Availability

The data that support the findings of this study are available from the corresponding authors upon reasonable request.

## References

[1] Radostits O. M. , Gay C. C. , Hinchcliff K. W. , and Constable P. D. , Veterinary Medicine: A Textbook of the Diseases of Cattle, Horses, Sheep, Pigs and Goats, 2007, 10th edition, Saunders Elsevier.

[2] Sharifiyazdi H. , Jafari S. , Ghane M. , Nazifi S. , and Sanati A. , Genetic Characterization and Phylogenetic Analysis of Hemotrophic Mycoplasmas in Camel (*Camelus dromedarius*), Comparative Clinical Pathology. (2018) 27, no. 3, 789–794, 10.1007/s00580-018-2666-9.

[3] Perera B. and Oswin M. A. , Livestock Production-Current Status in South and South-East Asia, Future Directions and Priority Areas for Research, Animal Production and Health Newsletter; Joint FAO/IAEA Division of Nuclear Techniques in Food and Agriculture, Animal Production and Health Section: Vienna, Austria, 2014, 59, https://www.osti.gov/etdeweb/biblio/22190329.

[4] Soosaraei M. , Haghi M. M. , Etemadifar F. et al., Status of Anaplasma spp. Infection in Domestic Ruminants From Iran: A Systematic Review With Meta-Analysis, Parasite Epidemiology and Control. (2020) 11, 10.1016/j.parepi.2020.e00173. PMC7452112 32875131

[5] Kocan K. M. , de la Fuente J. , and Cabezas-Cruz A. , The Genus Anaplasma: New Challenges After Reclassification, Revue Scientifique et Technique (International Office of Epizootics). (2015) 34, no. 2, 577–586, 10.20506/rst.34.2.2381. 26601458

[6] Aktas M. , Altay K. , and Dumanli N. , Molecular Detection and Identification of *Anaplasma* and *Ehrlichia* Species in Cattle from Turkey, Ticks and Tick-Borne Diseases. (2011) 2, no. 1, 62–65, 10.1016/j.ttbdis.2010.01.001. 21771539

[7] Karlsen A. , Vojtek B. , Mojžišová J. , Prokeš M. , and Drážovská M. , Anaplasmosis in Animals, Folia Veterinaria. (2020) 64, no. 4, 17–26, 10.2478/fv-2020-0033.

[8] Kocan K. M. , de la Fuente J. , Blouin E. F. , and Garcia-Garcia J. C. , Anaplasma marginale (Rickettsiales: Anaplasmataceae): Recent Advances in Defining Host-Pathogen Adaptations of a Tick-Borne Rickettsia, Parasitology. (2010) 137, no. 9, 1185–1198. 10.1017/s0031182003004700 15938516

[9] Mubashir M. , Tariq M. , Khan M. S. et al., Review on Anaplasmosis in Different Ruminants, Zeugma Biological Science. (2022) 3, no. 2, 32–45.

[10] Windsor P. A. , Anaemia in Lambs Caused by Mycoplasma Ovis: Global and Australian Perspectives, Animals. (2022) 12, no. 11, 10.3390/ani12111372. PMC9179446 35681835

[11] Neitz W. O. , Eperythrozoonosis in Sheep, Onderstepoort Journal of Veterinary Science and Animal Industry. (1937) 9.

[12] Abed F. A. and Alsaad K. M. , Clinical, Hematological and Diagnostic Studies of Hemomycoplasma Infection (Mycoplasma ovis) in Sheep of Basrah Governorate, Basrah Journal of Veterinary Research. (2017) 16, no. 2, 284–301.

[13] Paul B. T. , Jesse F. F. A. , Chung E. L. T. et al., Review of Clinical Aspects, Epidemiology and Diagnosis of Haemotropic Mycoplasma ovis in Small Ruminants: Current Status and Future Perspectives in Tropics Focusing on Malaysia, Tropical Animal Health and Production. (2020) 52, no. 6, 2829–2844, 10.1007/s11250-020-02357-9. 32712811 PMC7382646

[14] Machado C. A. L. , Vidotto O. , Conrado F. O. et al., Mycoplasma ovis Infection in Goat Farms from Northeastern Brazil, Comparative Immunology, Microbiology and Infectious Diseases. (2017) 55, 1–5, 10.1016/j.cimid.2017.08.004. 29127988

[15] Urie N. J. , Highland M. A. , Knowles D. P. , Branan M. A. , Herndon D. R. , and Marshall K. L. , Mycoplasma ovis Infection in Domestic Sheep (*Ovis aries*) in the United States: Prevalence, Distribution, Associated Risk Factors, and Associated Outcomes, Preventive Veterinary Medicine. (2019) 171, 10.1016/j.prevetmed.2019.104750. 31472359

[16] Wang X. , Cui Y. , Zhang Y. et al., Molecular Characterization of Hemotropic Mycoplasmas (Mycoplasma ovis and ‘Candidatus Mycoplasma Haemovis’) in Sheep and Goats in China, BMC Veterinary Research. (2017) 13, 1–8, 10.1186/s12917-017-1062-z. 28549435 PMC5446696

[17] Thlama P. B. , Abdullah J. F. F. , Juriah K. , Teik C. E. L. , Azlan C. , and Azmi M. L. , Further Insights into the Pathogenic Mechanisms of Haemotropic Mycoplasma ovis, Tropical Life Sciences Research. (2024) 35, no. 3. 10.21315/tlsr2024.35.3.15 PMC11507977 39464669

[18] Rahman M. , Faruque M. R. , Rahman M. M. , and Chowdhury M. Y. , Epidemiology and Molecular Detection of Anaplasma spp. in Goats from Chattogram District, Bangladesh, Veterinary Medicine and Science. (2022) 8, no. 3, 1240–1249, 10.1002/vms3.775. 35218684 PMC9122420

[19] Rosso F. , Tagliapietra V. , Baráková I. et al., Prevalence and Genetic Variability of Anaplasma phagocytophilum in Wild Rodents from the Italian Alps, Parasites & Vectors. (2017) 10, 1–8, 10.1186/s13071-017-2221-6. 28615038 PMC5471728

[20] Razzaq F. , Khosa T. , Ahmad S. et al., Prevalence of Anaplasma phagocytophilum in Horses From Southern Punjab (Pakistan), Tropical Biomedicine. (2015) 32, 233–239. 26691251

[21] Li H. , Zheng Y. C. , Ma L. et al., Human Infection With a Novel Tick-Borne Anaplasma Species in China: a Surveillance Study, The Lancet Infectious Diseases. (2015) 15, no. 6, 663–670, 10.1016/s1473-3099(15)70051-4. 25833289

[22] Peng Y. , Wang K. , Zhao S. et al., Detection and Phylogenetic Characterization of Anaplasma Capra: An Emerging Pathogen in Sheep and Goats in China, Frontiers in Cellular and Infection Microbiology. (2018) 8, 10.3389/fcimb.2018.00283. PMC6126426 30214896

[23] Yang J. , Han R. , Niu Q. et al., Occurrence of Four Anaplasma Species with Veterinary and Public Health Significance in Sheep, Northwestern China, Ticks and Tick-Borne Diseases. (2018) 9, no. 1, 82–85, 10.1016/j.ttbdis.2017.10.005. 29037826

[24] Lin Z. T. , Ye R. Z. , Liu J. Y. et al., Epidemiological and Phylogenetic Characteristics of Emerging Anaplasma Capra: A Systematic Review with Modeling Analysis, Infection, Genetics and Evolution, 2023, 115, 10.1016/j.meegid.2023.105510. 37778674

[25] Gholipoor Z. , Khazan H. , Azargashb E. , Youssefi M. R. , and Rostami A. , Prevalence and Risk Factors of Intestinal Parasite Infections in Mazandaran Province, North of Iran, Clinical Epidemiology and Global Health. (2020) 8, no. 1, 17–20, 10.1016/j.cegh.2019.05.010.

[26] Hoelzle K. , Winkler M. , Kramer M. M. , Wittenbrink M. M. , Dieckmann S. M. , and Hoelzle L. E. , Detection of Candidatus Mycoplasma Haemobos in Cattle with Anaemia, The Veterinary Journal. (2011) 187, no. 3, 408–410, 10.1016/j.tvjl.2010.01.016. 20188610

[27] Teja M. M. S. , Mamatha G. S. , Lakkundi J. N. , Chandranaik B. M. , Murthy C. M. K. , and Gomes A. R. , Multiplex PCR for Detection of *Anaplasma marginale*, *A. bovis* and *A. platys* in Cattle, Journal of Parasitic Diseases. (2023) 47, no. 3, 659–663, 10.1007/s12639-023-01606-6. 37520189 PMC10382415

[28] Galon E. M. , Ybañez R. H. , Macalanda A. M. et al., First Molecular Identification of Babesia, Theileria, and Anaplasma in Goats from the Philippines, Pathogens. (2022) 11, no. 10, 10.3390/pathogens11101109. PMC9612162 36297166

[29] Tamura K. , Stecher G. , and Kumar S. , MEGA11: Molecular Evolutionary Genetics Analysis Version 11, Molecular Biology and Evolution. (2021) 38, no. 7, 3022–3027, 10.1093/molbev/msab120. 33892491 PMC8233496

[30] Hosseini-Vasoukolaei N. , Oshaghi M. A. , hayan S. P. et al., Anaplasma Infection in Ticks, Livestock and Human in Ghaemshahr, Mazandaran Province, Iran, Journal of Arthropod-Borne Diseases. (2014) 8, no. 2. PMC4478432 26114134

[31] Kalantari M. , Sharifiyazdi H. , Ghane M. , and Nazifi S. , The Occurrence of Hemotropic Mycoplasma Ovis-Like Species in Horses, Preventive Veterinary Medicine. (2020) 175, 10.1016/j.prevetmed.2019.104877. 31896506

[32] Alekish M. O. and Ismail Z. B. , Relationship Between Certain Serum Biochemical Values and Serostatus Against Anaplasma marginale in Dairy Cows, Veterinary World. (2019) 12, no. 11. 10.14202/vetworld.2019.1858-1861 PMC6925038 32009766

[33] Ganguly A. , Maharana B. R. , Ganguly I. N. D. R. A. J. I. T. et al., Molecular Diagnosis and Haemato-Biochemical Changes in *Anaplasma marginale* Infected Dairy Cattle, Indian Journal of Animal Sciences. (2018) 88, no. 9, 989–993, 10.56093/ijans.v88i9.83538.

[34] Ahmadi-hamedani M. , Ahmadi-hamedani M. , Fathi E. , and Sani R. N. , Comparison of Selected Biochemical Parameters Between Naturally Infected and Non-Infected Goats with Anaplasma ovis, Comparative Clinical Pathology. (2014) 23, no. 4, 989–992, 10.1007/s00580-013-1730-8.

[35] Khaki Z. , Yasini S. P. , and Jalali S. , A Survey of Biochemical and Acute Phase Proteins Changes in Sheep Experimentally Infected With *Anaplasma ovis* , Asian Pacific Journal of Tropical Biomedicine. (2018) 8, no. 12, 565–570, 10.4103/2221-1691.248092.

[36] Messick J. B. , Hemotrophic Mycoplasmas (Hemoplasmas): A Review and New Insights into Pathogenic Potential, Veterinary Clinical Pathology. (2004) 33, no. 1, 2–13, 10.1111/j.1939-165x.2004.tb00342.x. 15048620

[37] Maggi R. G. , Compton S. M. , Trull C. L. , Mascarelli P. E. , Mozayeni B. R. , and Breitschwerdt E. B. , Infection with Hemotropic Mycoplasma Species in Patients With or Without Extensive Arthropod or Animal Contact, Journal of Clinical Microbiology. (2013) 51, no. 10, 3237–3241, 10.1128/jcm.01125-13. 23863574 PMC3811635

[38] Aguirre D. H. , Thompson C. , Neumann R. D. , Salatin A. O. , Gaido A. B. , and Torioni de Echaide S. , Clinical Mycoplasmosis Outbreak due to *Mycoplasma ovis* in Sheep From Shalta, Argentina. Clinical, Microbiological and Molecular Diagnosis, Revista Argentina de Microbiología. (2009) 41, no. 4, 212–214. 20085183

[39] Hornok S. , Hajtós I. , Meli M. et al., First Molecular Identification of Mycoplasma ovis and ‘*Candidatus M. Haemoovis*’ From Goat, with Lack of Haemoplasma PCR-Positivity in Lice, Acta Veterinaria Hungarica. (2012) 60, no. 3, 355–360, 10.1556/avet.2012.030. 22903080

[40] Sykes J. E. , Lindsay L. L. , Maggi R. G. , and Breitschwerdt E. B. , Human Coinfection with Bartonella henselae and Two Hemotropic Mycoplasma Variants Resembling Mycoplasma ovis, Journal of Clinical Microbiology. (2010) 48, no. 10, 3782–3785, 10.1128/jcm.01029-10. 20702675 PMC2953074

[41] Jalali S. M. , Khaki Z. , Kazemi B. et al., Molecular Detection and Identification of Anaplasma Species in Sheep from Ahvaz, Iran, Iranian Journal of Veterinary Research. (2013) 14, no. 1, 50–56.

[42] Khaki Z. , Jalali S. M. , Kazemi B. , Jalali M. R. , and Yasini S. P. , A Study of Hematological Changes in Sheep Naturally Infected with Anaplasma spp. and Theileria Ovis: Molecular Diagnosis, 2015.

[43] Razmi G. R. , Dastjerdi K. , Hossieni H. , Naghibi A. , Barati F. , and Aslani M. R. , An Epidemiological Study on Anaplasma Infection in Cattle, Sheep, and Goats in Mashhad Suburb, Khorasan Province, Iran, Annals of the New York Academy of Sciences. (2006) 1078, no. 1, 479–481, 10.1196/annals.1374.089. 17114758

[44] Noaman V. and Shayan P. , Comparison of Microscopy and PCR-RFLP for Detection of Anaplasma marginale in Carrier Cattle, Iranian Journal of Microbiology. (2010) 2, no. 2. PMC3279773 22347555

[45] Zadeh S. S. , Fathi S. , Dehaghi M. M. , Asl E. N. , and Nezhad H. A. , Survey of Theileria annulata and Anaplasma marginale in Cattle in Kerman Area, Southeast of Iran, Scientia Parasitologica. (2011) 12, 61–66.

[46] Ameen K. A. H. , Abdullah B. A. , and Abdul-Razaq R. A. , Seroprevalence of Babesia bigemina and Anaplasma marginale in Domestic Animals in Erbil, Iraq, Iraqi Journal of Veterinary Sciences. (2012) 26, no. Suppl. III, 109–114, 10.33899/ijvs.2012.168747.

[47] Jafarbekloo A. , Bakhshi H. , Faghihi F. et al., Molecular Detection of Anaplasma and Ehrlichia Infection in Ticks in Borderline of Iran-Afghanistan, Journal of Biomedical Science and Engineering. (2014) 7, no. 11.

[48] Kaewmongkol G. , Lukkana N. , Yangtara S. et al., Association of Ehrlichia Canis, Hemotropic Mycoplasma spp. and Anaplasma platys and Severe Anemia in Dogs in Thailand, Veterinary Microbiology. (2017) 201, 195–200, 10.1016/j.vetmic.2017.01.022. 28284610

[49] Rar V. A. , Livanova N. N. , Panov V. V. et al., Genetic Diversity of Anaplasma and Ehrlichia in the Asian Part of Russia, Ticks and Tick-borne Diseases. (2010) 1, no. 1, 57–65, 10.1016/j.ttbdis.2010.01.002. 21771512

[50] Atif F. A. , Prevalence of Tick-Borne Diseases in Punjab (Pakistan) and Hematological Profile of Anaplasma marginale Infection in Indigenous and Crossbred Cattle, Pakistan Journal of Science. (2012) 64, no. 1.

[51] Kabay M. J. , Richards R. B. , and Ellis T. E. , A Cross-Sectional Study to Show Eperythrozoon ovis Infection is Prevalent in Western Australian Sheep Farms, Australian Veterinary Journal. (1991) 68, no. 5, 170–173, 10.1111/j.1751-0813.1991.tb03172.x. 1883295

[52] Mason R. W. , Corbould A. , and Statham P. , A Serological Survey of Eperythrozoon ovis in Goats and Sheep in Tasmania, 1989, 122–123. 10.1111/j.1751-0813.1989.tb09767.x 2658946

[53] Rjeibi M. R. , Darghouth M. A. , Omri H. , Souidi K. , Gharbi M. , and Rekik M. , First Molecular Isolation of Mycoplasma ovis from Small Ruminants in North Africa, Onderstepoort Journal of Veterinary Research. (2015) 82, no. 1, 1–5, 10.4102/ojvr.v82i1.912. PMC6238789 26244681

[54] Shompole S. , Waghela S. D. , Rurangirwa F. R. , and McGuire T. C. , Cloned DNA Probes Identify Anaplasma ovis in Goats and Reveal a High Prevalence of Infection, Journal of Clinical Microbiology. (1989) 27, no. 12, 2730–2735, 10.1128/jcm.27.12.2730-2735.1989. 2592538 PMC267118

[55] Ndung’u L. W. , Aguirre C. , Rurangirwa F. R. et al., Detection of *Anaplasma ovis* Infection in Goats by Major Surface Protein 5 Competitive Inhibition enzyme-linked Immunosorbent Assay, Journal of Clinical Microbiology. (1995) 33, no. 3, 675–679, 10.1128/jcm.33.3.675-679.1995. 7538510 PMC228012

[56] Kubelová M. and Široký J. M. P. , Theileria, Babesia, and Anaplasma Detected by PCR in Ruminant Herds at Bié Province, Angola, Parasite. (2012) 19, no. 4, 417–422, 10.1051/parasite/2012194417. 23193527 PMC3671455

[57] Shiri A. , Kheirandish F. , Sazmand A. , Kayedi M. H. , and Hosseini-Chegeni A. , Molecular Identification of Hemoparasites in Ixodid Ticks in Iran, Veterinary Parasitology: Regional Studies and Reports. (2024) 47, 10.1016/j.vprsr.2023.100967. 38199703

[58] Kolo A. , Anaplasma Species in Africa—A Century of Discovery: A Review on Molecular Epidemiology, Genetic Diversity, and Control, Pathogens. (2023) 12, no. 5, 10.3390/pathogens12050702. PMC10222256 37242372

[59] Lin Z. T. , Du L. F. , Zhang M. Z. et al., Genomic Characteristics of Emerging Intraerythrocytic Anaplasma Capra and High Prevalence in Goats, China, Emerging Infectious Diseases. (2023) 29, no. 9, 10.3201/eid2909.230131. PMC10461651 37610104

[60] Adogla-Bessa and Aganga A. A. , Responses of Tswana Goats to Various Lengths of Water Deprivation, South African Journal of Animal Science. (2000) 30, no. 1, 87–91, 10.4314/sajas.v30i1.3883.

[61] Smith R. C. , Myers S. , Sundstrom K. D. et al., Detection of Anaplasma Bovis-like Agent in the Southcentral United States, Ticks and Tick-Borne Diseases. (2024) 15, no. 6, 10.1016/j.ttbdis.2024.102411. 39550988

[62] Barbosa I. C. , André M. R. , do Amaral R. B. et al., Anaplasma marginale in Goats from a Multispecies Grazing System in Northeastern Brazil, Ticks and Tick-borne Diseases. (2021) 12, no. 1, 10.1016/j.ttbdis.2020.101592. 33099171

[63] Alessandra T. and Santo C. , Tick-Borne Diseases in Sheep and Goats: Clinical and Diagnostic Aspects, Small Ruminant Research. (2012) 106, S6–S11, 10.1016/j.smallrumres.2012.04.026.

[64] Yeruham I. , Hadani A. , and Galker F. , Some Epizootiological and Clinical Aspects of Ovine Babesiosis Caused by Babesia ovis—A Review, Veterinary Parasitology. (1998) 74, no. 2-4, 153–163, 10.1016/s0304-4017(97)00143-x. 9561703

[65] Neimark H. , Hoff B. , and Ganter M. , *Mycoplasma ovis* Comb. Nov.(Formerly Eperythrozoon Ovis), an Epierythrocytic Agent of Haemolytic Anaemia in Sheep and Goats, International Journal of Systematic and Evolutionary Microbiology. (2004) 54, no. 2, 365–371, 10.1099/ijs.0.02858-0. 15023944

[66] Mongruel A. C. B. , Spanhol V. C. , Valente J. D. M. et al., Survey of Vector-Borne and Nematode Parasites Involved in the Etiology of Anemic Syndrome in Sheep from Southern Brazil, Revista Brasileira de Parasitologia Veterinaria. (2020) 29, no. 3, 10.1590/s1984-29612020062. 32935770

[67] Ahmadi-hamedani M. , Khaki Z. , Rahbari S. , and Ahmadi-hamedani M. A. , Hematological Profiles of Goats Naturally Infected with Anaplasma ovis in North and Northeast Iran, Comparative Clinical Pathology. (2012) 21, no. 6, 1179–1182, 10.1007/s00580-011-1257-9.

[68] Ranjbar R. , Anjomruz M. , Enayati A. A. , Khoobdel M. , Rafinejad A. , and Rafinejad J. , Anaplasma Infection in Ticks in Southeastern Region of Iran, Journal of Arthropod-Borne Diseases. (2020) 14, no. 2, 10.18502/jad.v14i2.3730. PMC7738930 33365340

[69] Rahravani M. , Moravedji M. , Mostafavi E. et al., Clinical, Hematological and Molecular Evaluation of Piroplasma and Anaplasma Infections in Small Ruminants and Tick Vectors from Kurdistan Province, Western Iran, Research in Veterinary Science. (2023) 159, 44–56, 10.1016/j.rvsc.2023.03.025. 37080001

[70] Duscher G. G. , Leschnik M. , Fuehrer H. P. , and Joachim A. , Wildlife Reservoirs for Vector-Borne Canine, Feline and Zoonotic Infections in Austria, International Journal of Parasitology: Parasites and Wildlife. (2015) 4, no. 1, 88–96, 10.1016/j.ijppaw.2014.12.001. PMC4356739 25830102

[71] Plowright R. K. , Parrish C. R. , McCallum H. et al., Pathways to Zoonotic Spillover, Nature Reviews Microbiology. (2017) 15, no. 8, 502–510, 10.1038/nrmicro.2017.45. 28555073 PMC5791534

[72] Daszak P. , Cunningham A. A. , and Hyatt A. D. , Emerging Infectious Diseases of Wildlife--Threats to Biodiversity and Human Health, Science. (2000) 287, no. 5452, 443–449, 10.1126/science.287.5452.443. 10642539

[73] Cunningham A. A. , Daszak P. , and Wood J. L. , One Health, Emerging Infectious Diseases and Wildlife: Two Decades of Progress?, Philosophical Transactions of the Royal Society B: Biological Sciences. (2017) 372, no. 1725, 10.1098/rstb.2016.0167. PMC5468692 28584175

[74] Titcomb G. , Allan B. F. , Ainsworth T. et al., Interacting Effects of Wildlife Loss and Climate on Ticks and tick-borne Disease, Proceedings of the Royal Society B: Biological Sciences. (2017) 284, no. 1862, 10.1098/rspb.2017.0475. PMC5597820 28878055

[75] Beyer A. R. and Carlyon J. A. , Of Goats and Men: Rethinking Anaplasmoses as Zoonotic Infections, Lancet Infectious Diseases. (2015) 15, no. 6, 619–620, 10.1016/s1473-3099(15)70097-6. 25833288 PMC4881300

[76] Amer S. , Kim S. , Yun Y. , and Na K. J. , Novel Variants of the Newly Emerged Anaplasma Capra from Korean Water Deer (Hydropotes inermis Argyropus) in South Korea, Parasites & Vectors. (2019) 12, 1–9, 10.1186/s13071-019-3622-5. 31345253 PMC6659236

[77] Sato M. , Nishizawa I. , Fujihara M. , Nishimura T. , Matsubara K. , and Harasawa R. , Phylogenetic Analysis of the 16S Rrna Gene of Anaplasma Species Detected From Japanese Serows (*Capricornis crispus*), Journal of Veterinary Medical Science. (2009) 71, no. 12, 1677–1679, 10.1292/jvms.001677. 20046041

[78] Yang J. , Liu Z. , Niu Q. et al., A Novel Zoonotic Anaplasma Species is Prevalent in Small Ruminants: Potential Public Health Implications, Parasites & Vectors. (2017) 10, 1–6, 10.1186/s13071-017-2182-9. 28558749 PMC5450374

[79] Grandi G. , Aspán A. , Pihl J. et al., Detection of Tick-Borne Pathogens in Lambs Undergoing Prophylactic Treatment Against Ticks on Two Swedish Farms, Frontiers in Veterinary Science. (2018) 5, 10.3389/fvets.2018.00072. PMC5911771 29713635

[80] Seo M. G. , Ouh I. O. , Lee H. , Geraldino P. J. L. , Rhee M. H. , and Kwak O. D. K.D. , Differential Identification of Anaplasma in Cattle and Potential of Cattle to Serve as Reservoirs of *Anaplasma Capra*, an Emerging tick-borne Zoonotic Pathogen, Veterinary Microbiology. (2018) 226, 15–22, 10.1016/j.vetmic.2018.10.008. 30389039

